# Supplementary material for: Joint profiling of DNA and proteins in single cells to dissect genotype-phenotype associations in leukemia
Source: Nat Commun. 2021 Mar 11;12:1583. doi: 10.1038/s41467-021-21810-3 (PMC7952600; doi:10.1038/s41467-021-21810-3)
Supplement: Supplementary file 1 — Supplementary Information [file 41467_2021_21810_MOESM1_ESM.pdf]

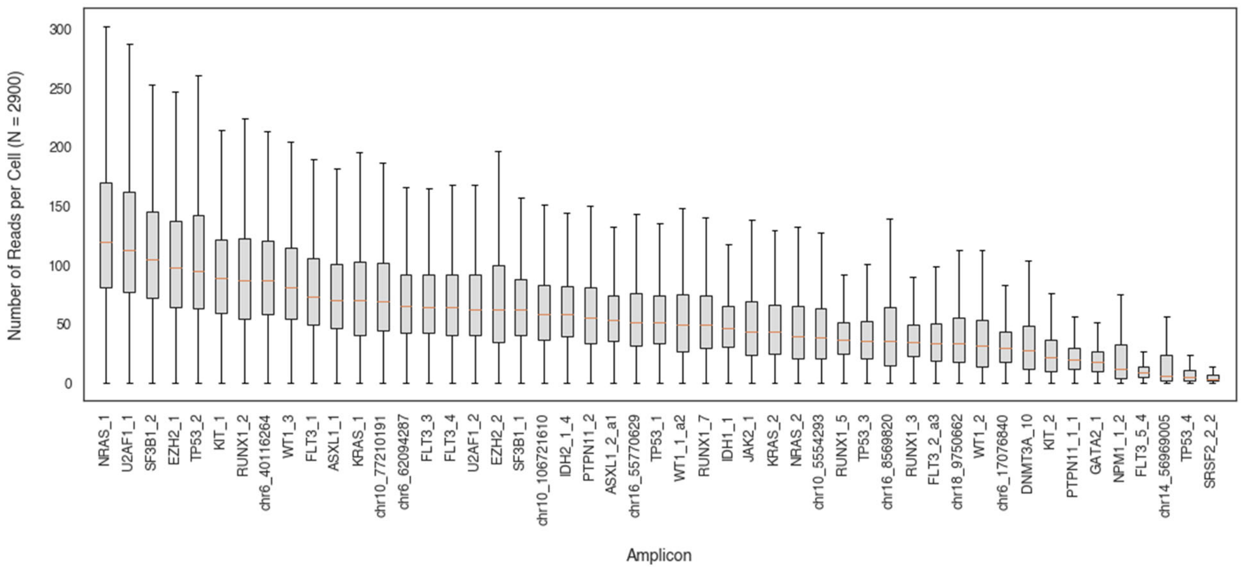

Supplementary Figure 1: Reads per DNA target for the 3-cell control experiment. Boxplot indicating the distribution of reads per cell (n = 2,900 single cells) for each of the 49 DNA panel targets in the cell line control experiment. DNA targets are sorted in descending order by median number of reads per cell (orange bar). The grey box represents the interquartile range. The lower and upper whiskers are drawn extending from the first and third quartiles, respectively, to the nearest observed point falling within 1.5 times the interquartile range.

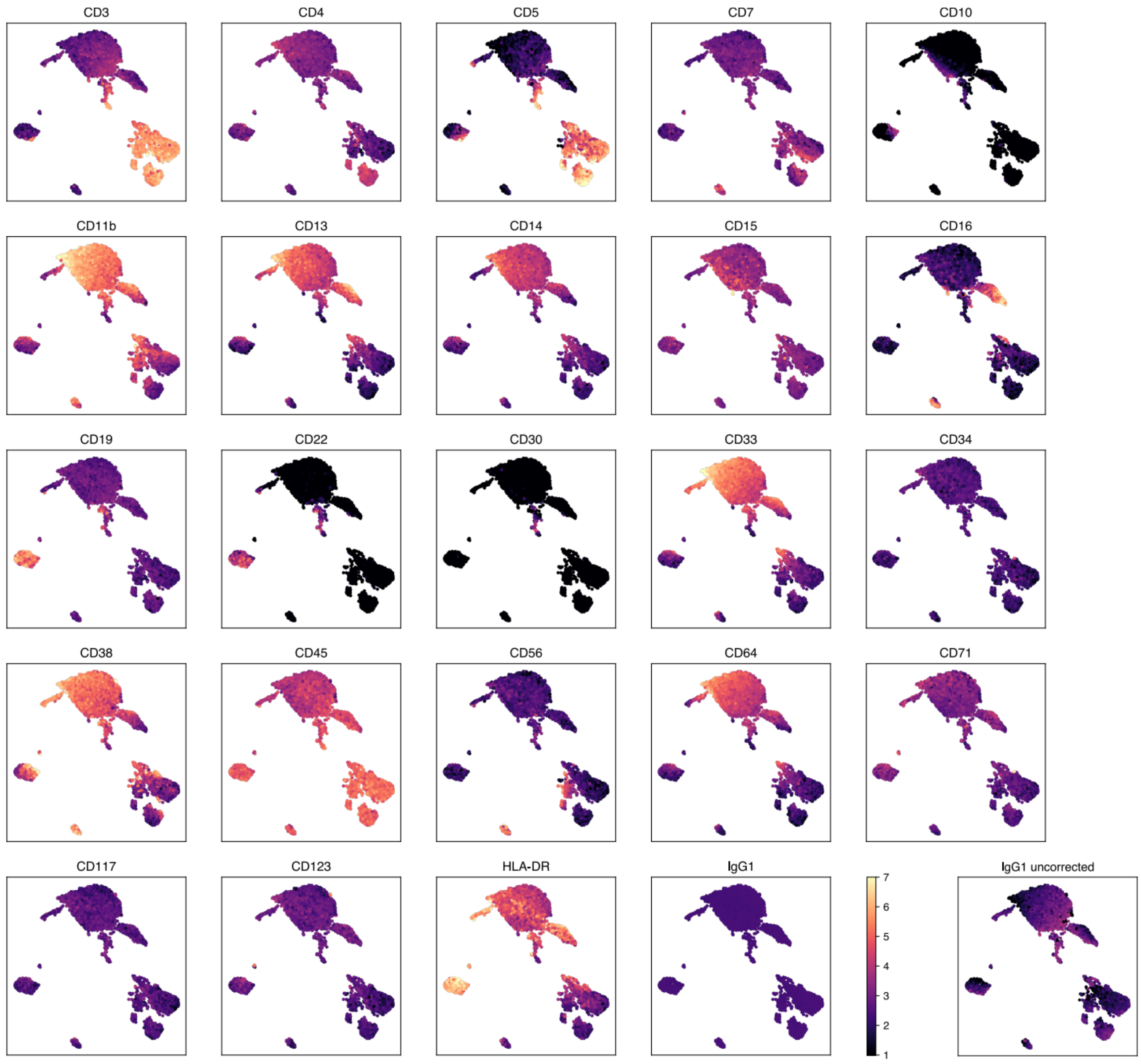

Supplementary Figure 2: Antibody signal distribution in the PBMC control.
Corrected antibody counts (log scale with base *e*) for each cell and antibody are given as a heatmap using the UMAP coordinates from Figure 2. Heatmap values are clipped at 1 and 7. Since IgG1 signal was used in the antibody correction as a co-regressor together with other size factors (see Methods), the residual count values are uniform. Therefore, the non-regressed log-transformed IgG1 counts are provided in an additional panel (lower-right).

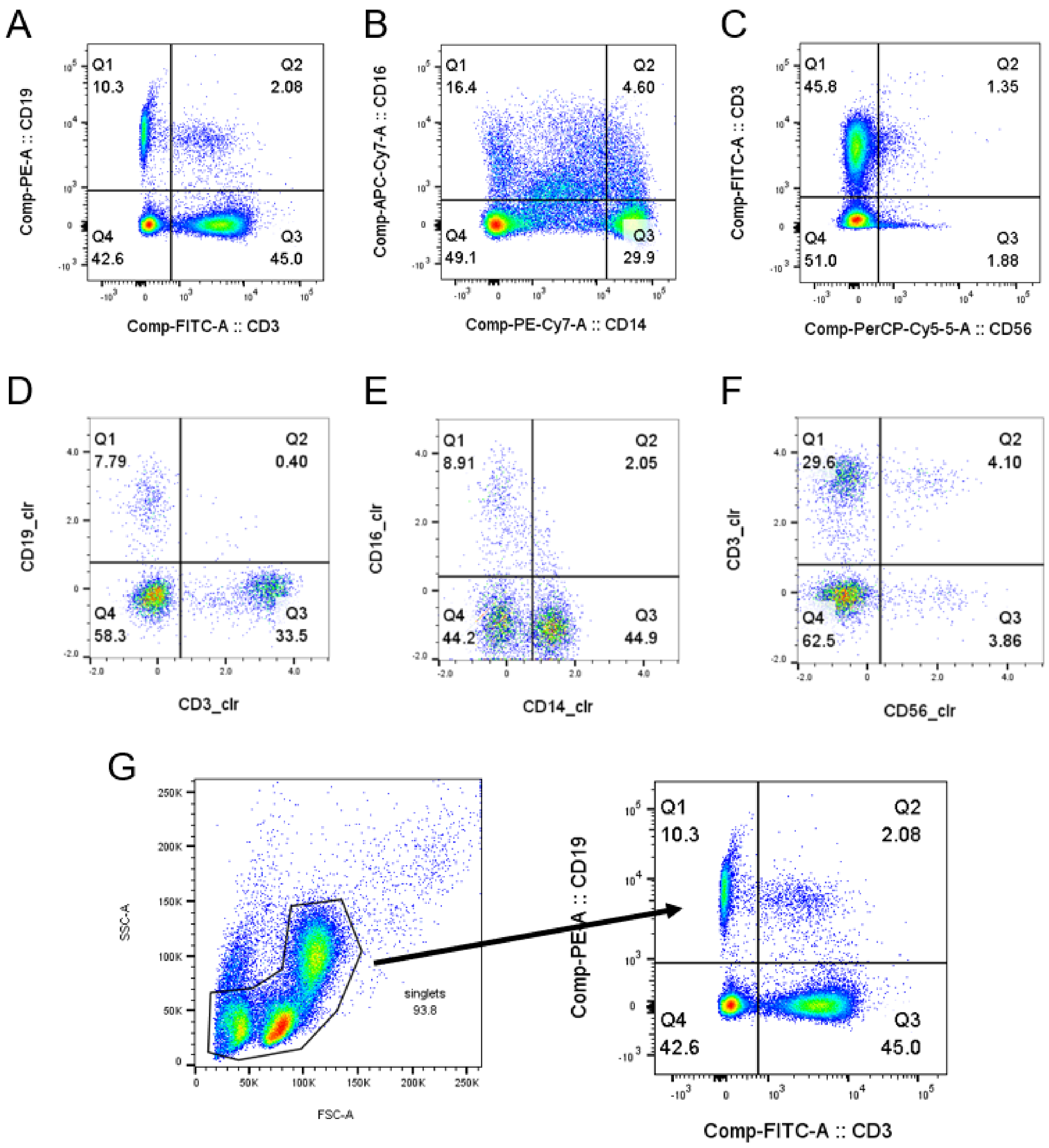

Supplementary Figure 3: Comparison of DAb-seq data to flow cytometry for healthy PBMCs.

(A, B, C) Flow cytometry results using five hematopoietic markers (CD3, CD14, CD16, CD19, CD56) to discriminate blood cell populations in healthy PBMCs. **(D, E, F)** DAb-seq results for the same PBMC sample across the five markers. Antibody signal is expressed as the centered log ratio for each cell. **(G)** Representative gating strategy for the flow cytometry experiments. A common cell singlet gate is first derived from the SSC/FSC channel. This population is then analyzed in each fluorescence channel.

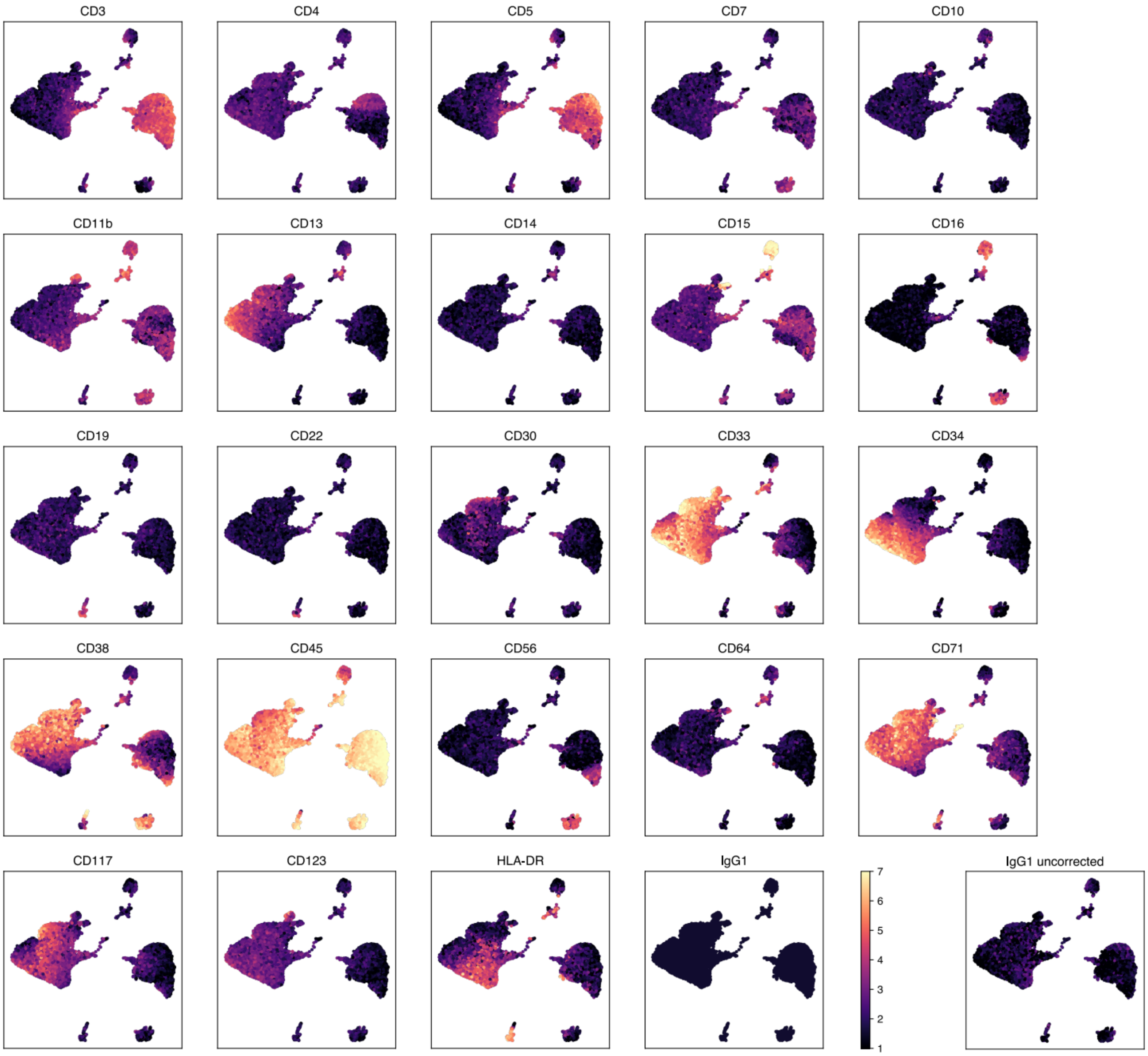

Supplementary Figure 4: Antibody signal UMAP for Patient #1 (gemtuzumab treated). Corrected antibody counts (log scale with base *e*) for each cell and antibody are given as a heatmap using the UMAP coordinates from Figure 3. Non-regressed log-transformed IgG1 counts are provided in an additional panel (lower-right).

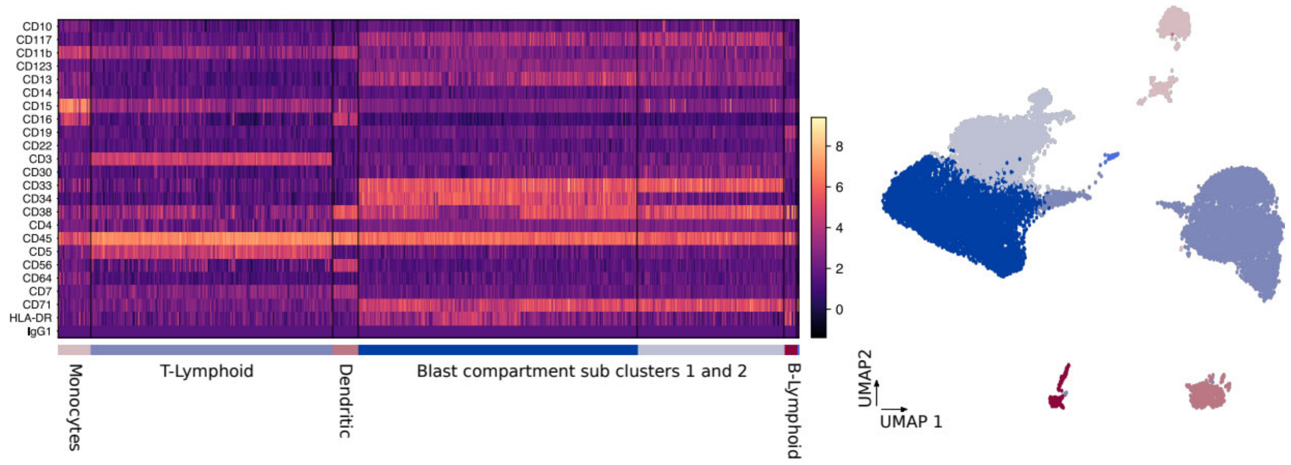

Supplementary Figure 5: Antibody count heatmap for Patient #1 (gemtuzumab treated). Corrected antibody counts (log scale with base *e*) are represented as a heatmap and sorted by the cell compartment clusters as described in the main text (Leiden clustering of the corrected antibody count matrix at a resolution factor of 0.4).

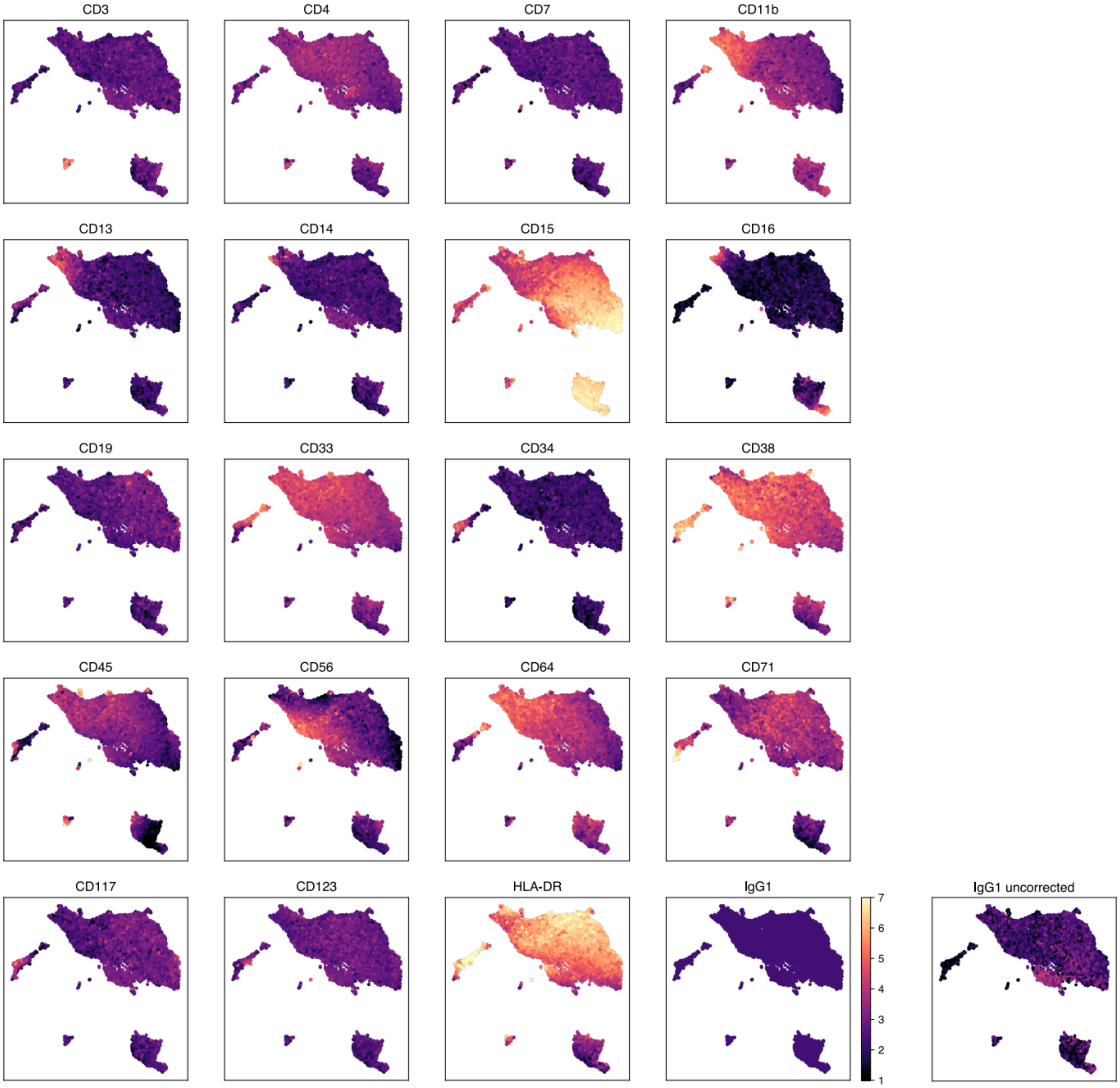

Supplementary Figure 6: Antibody signal UMAP for Patient #2 (pediatric patient). Corrected antibody counts (log scale with base *e*) for each cell and antibody are given as a heatmap using the UMAP coordinates from Figure 4. Non-regressed log-transformed IgG1 counts are provided in an additional panel (lower-right).

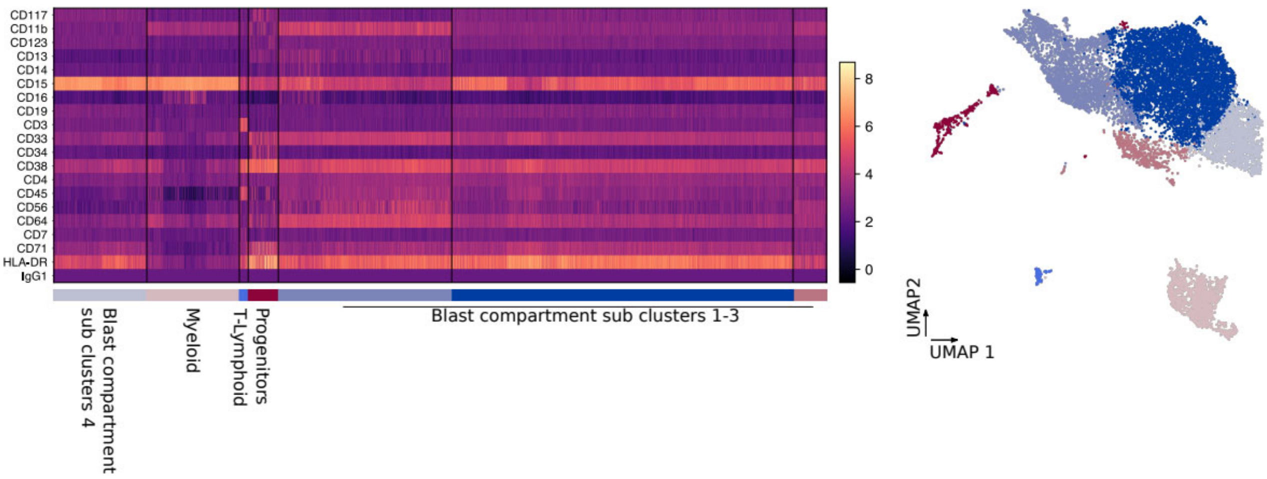

Supplementary Figure 7: Antibody count heatmap for Patient #2 (pediatric patient). Corrected antibody counts (log scale with base *e*) are represented as a heatmap and sorted by the cell compartment clusters as described in the main text (Leiden clustering of the corrected antibody count matrix at a resolution factor of 0.4). Blast subclusters 1-3 are not well separated.

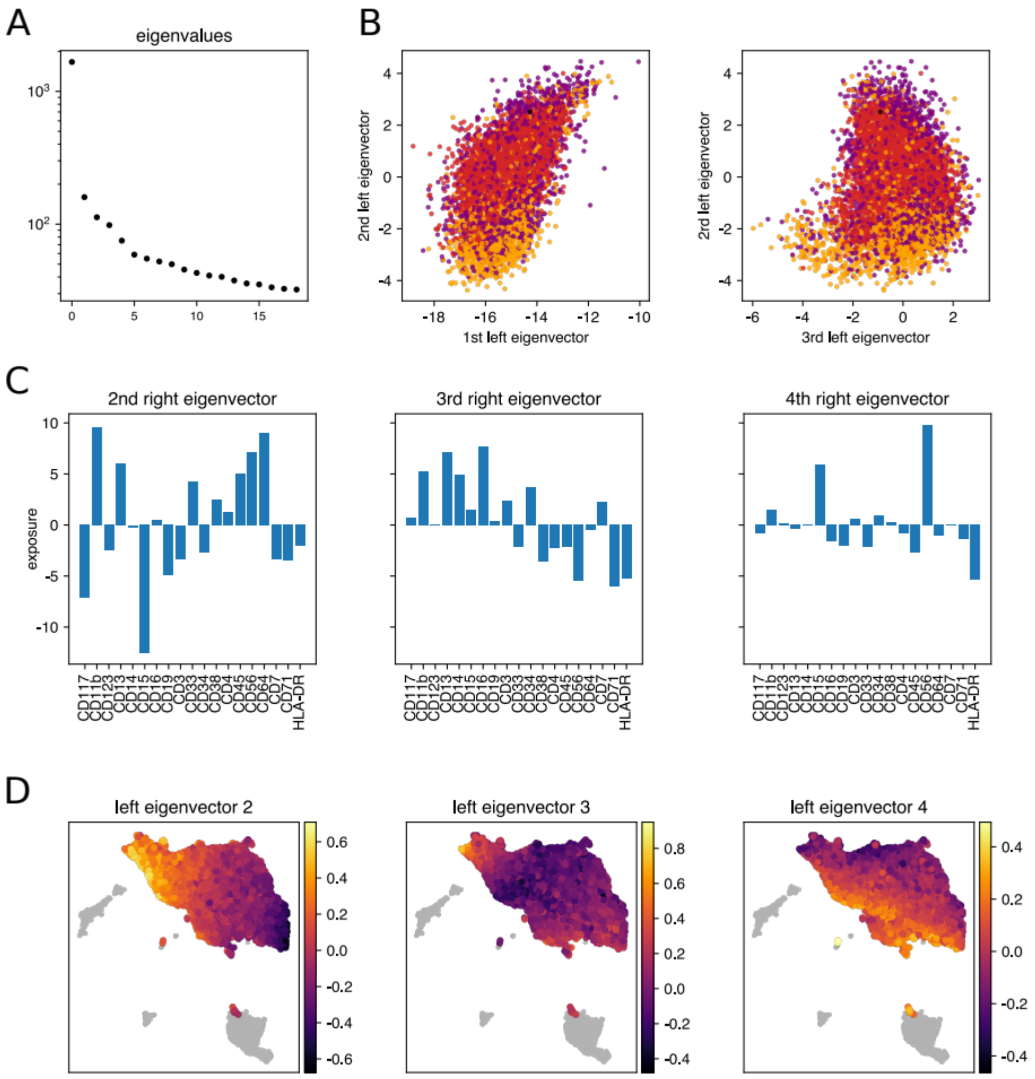

Supplementary Figure 8: Singular value decomposition of blast cell compartment for Patient #2 (pediatric patient).

Continuous trends are not easily identified by clustering. To discover such gradients in the antibody expression of the blast cells, singular value decomposition was calculated for the log-transformed and corrected cell by antibody counts matrix (**$A = U * s * V^T$**) (blast cells only. No mean centering or additional normalization was performed). **(A)** Ordered singular values are given. **(B)** Scatter plot of individual cells in coordinates of the first three left singular vectors (scaled by the corresponding singular value, that is: **$U_i * s_i$**). Colors are according to the genotype, same as in figure 4. **(C)** Second to fourth right singular vectors are given (again scaled by the corresponding singular value: **$s_i * V^T{}_i$**) which show the contribution of each antibody to the principal component. For example, CD15 and CD117 are anticorrelated with the second principal component (2nd left eigenvector), and CD11b, CD13, CD56 and CD64 are correlated. The first right eigenvector is not plotted. Because antibody counts are all positive numbers, the first principal component of the decomposition corresponds to the count offset and therefore carries no useful gradient information. **(D)** Scatter plot of cells in same UMAP coordinates as in figure 4. Each cell within the blast compartment is colored according to its 2nd, 3rd, or 4th left eigenvector value and highlights antibody count gradients within the blast compartment.

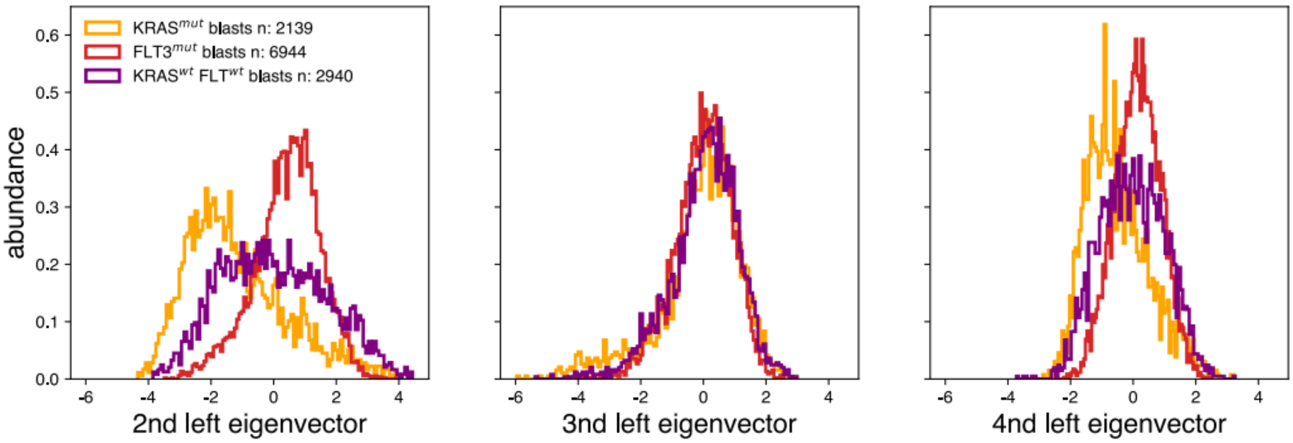

Supplementary Figure 9: Blast genotype distribution along the 2nd, 3rd, and 4th left eigenvectors for Patient #2 (pediatric patient).

Distribution of cells within the blast compartment along the 2nd, 3rd, and 4th left eigenvectors are shown. Histograms are plotted for each detected genotype separately. This shows that the genotype distribution within the blast compartments are biased toward a phenotypic cluster, but do not separate completely. In consequence, no FACS gating strategy can successfully deliver a genetically pure cell sample.

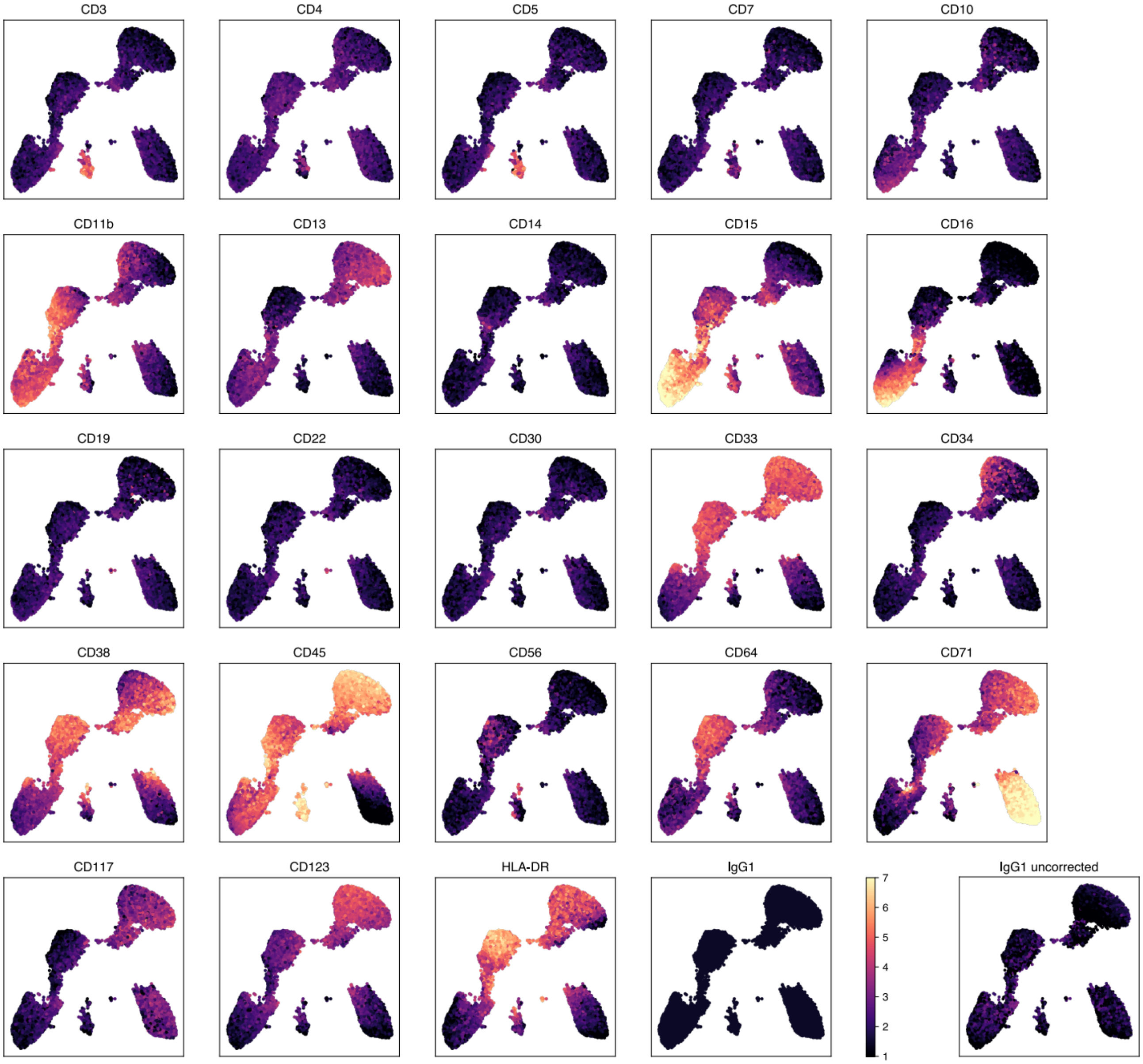

Supplementary Figure 10: Antibody signal UMAP for Patient #3 (FLT3 inhibitor treated). Corrected antibody counts (log scale with base *e*) for each cell and antibody are given as a heatmap using the UMAP coordinates from Figure 5. Non-regressed log-transformed IgG1 counts are provided in an additional panel (lower-right).

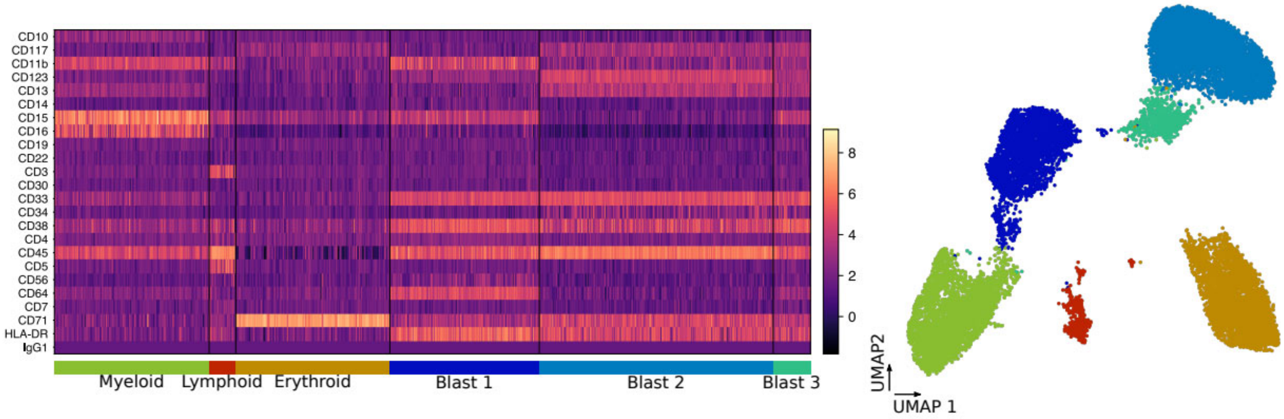

Supplementary Figure 11: Antibody count heatmap for Patient #3 (FLT3 inhibitor treated).

Corrected antibody counts (log scale with base *e*) are represented as a heatmap and sorted by the cell compartment clusters as described in the main text (Leiden clustering of the corrected antibody count matrix at a resolution factor of 0.4). Blast subclusters 1-3 are not well separated. Different lymphoid compartments are not labeled in the heatmap due to small cell numbers (e.g. B-lymphocytes and NK-lymphocytes), however, the clusters are visible in the UMAP representation.

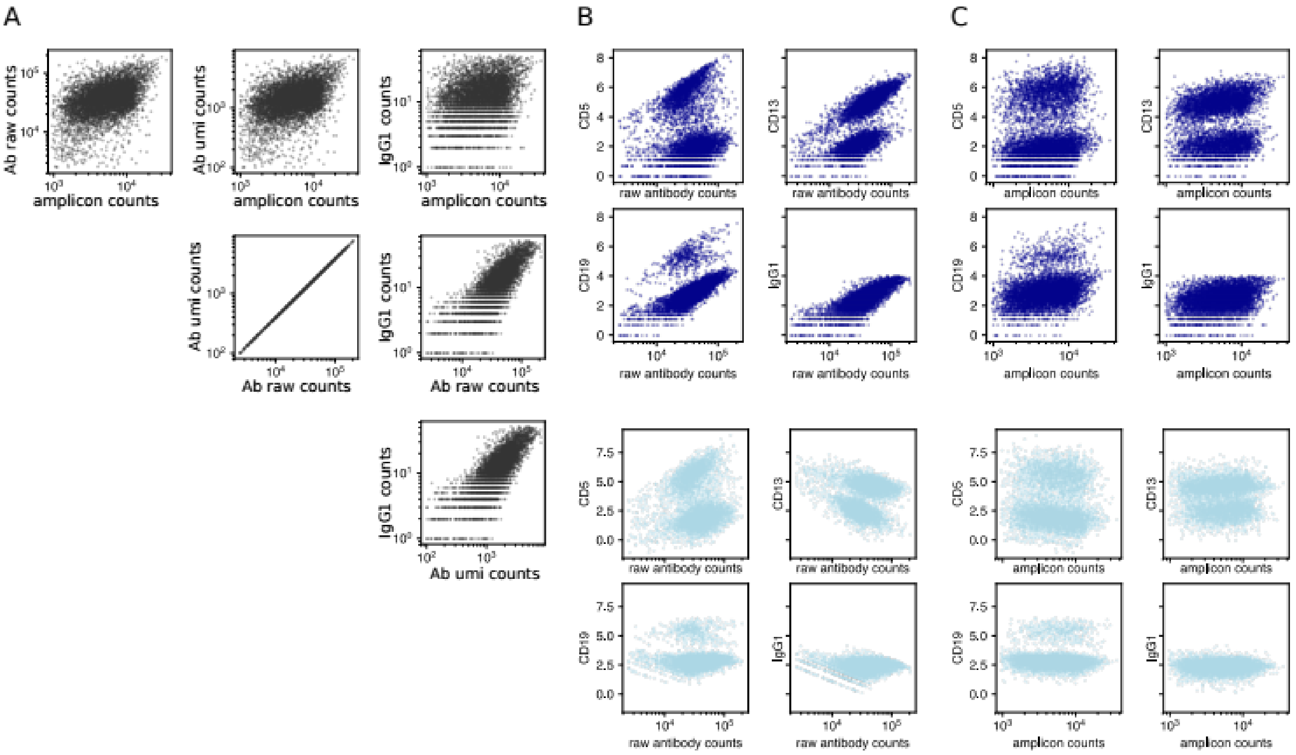

Supplementary Figure 12: Antibody count correction performance.
(A) Correlation among different cellular quality metrics (total antibody raw count, total antibody UMI count, amplicon count, total IgG1 count) are given as scatter plot for all cells in the PBMC control experiment. **(B)** For each cell in the PBMC experiment, raw antibody count (dark blue, top panel) or corrected antibody count (light blue, bottom panel) for four markers is plotted against the total raw antibody count per cell. Correlation in raw counts with total antibody count is clearly visible and present in the isotype control. Linear regression with the quality metrics as covariates reduces this dependency (light blue, bottom panel). **(C)** Same as (B) but plotted against total DNA panel read count. The correlation between total DNA panel count and individual antibody counts is weaker but nevertheless present.

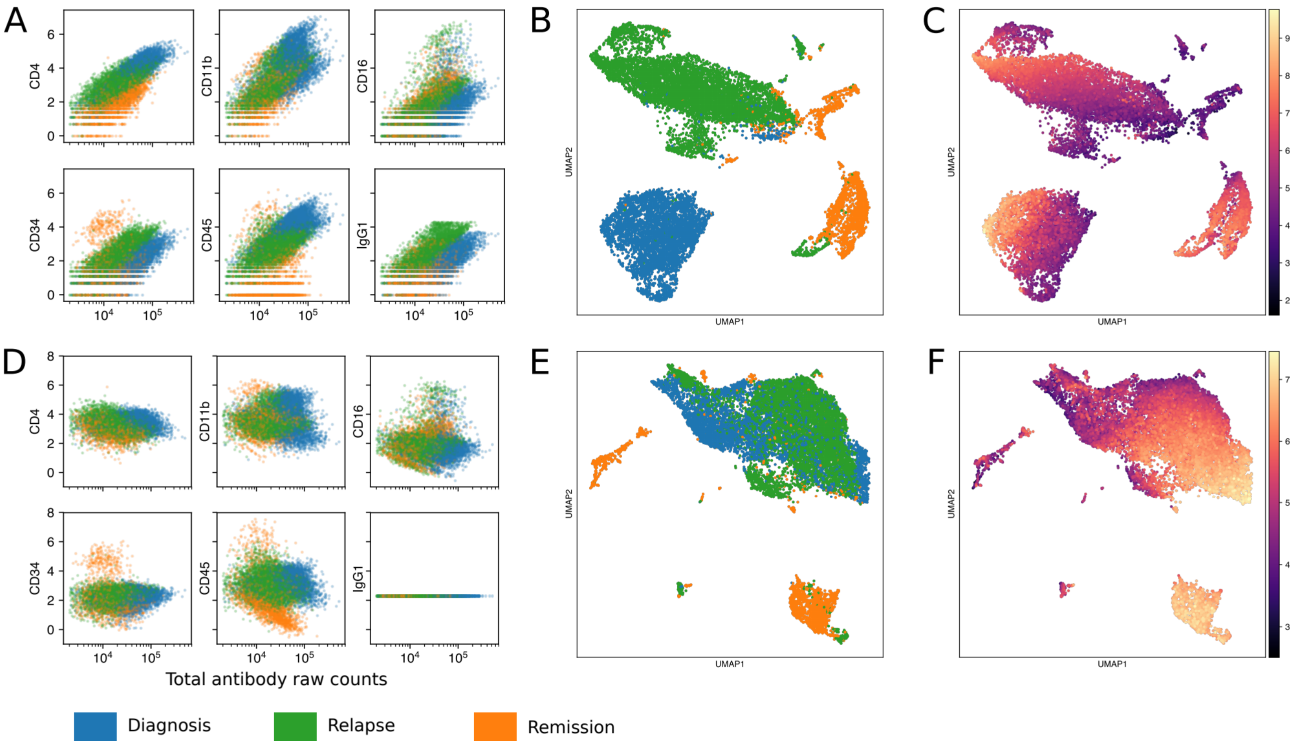

Supplementary Figure 13: Simultaneous antibody count regression corrects for batch effects.

(A) Six representative antibodies are plotted versus uncorrected total antibody counts for each cell in the Patient #2 (pediatric patient) series. Colors indicate sample timepoint. Batch effects are visible as an offset in the y-axis. **(B)** UMAP embedding of the uncorrected log transformed antibody counts is given. Color indicates sample timepoint. **(C)** Same UMAP embedding as in (B) but colored according to log(raw CD15 count). **(D)** Same as (A) after antibody count correction. Batch effect is reduced compared to (A). **(E)** Same as (B) but using corrected antibody counts to construct the UMAP embedding. After antibody correction, blast cells cluster together. **(F)** Same as (C) for corrected antibody counts. (E) and (F) correspond to the UMAP depicted in Figure 4.

Supplementary Table 1: DNA panel primer sequences

Reverse Primer 5' Adapter Sequence: GTCTCGTGGGCTCGGAGATGTGTATAAGAGACAG

#	Name	Foward Primer (5' to 3')	Reverse Primer (5' to 3') [5' adapter not shown]	Notes
1	ASXL1_1	CAGGACCCTCGCAGACATT*A*A*A	GGCAGTAGTTGTGTTCGCTGTA	
2	ASXL1_2_a1	CATGAGCCACCAAGCCC*T*A*A	TGTGAGTCTGGCACCACTTC	
3	DNMT3A_10	TTTGTGTCGCTACCTCAGT*T*T*G	GGTCCTGCTGTGTGGTTAGAC	
4	EZH2_1	TGCAAATTCAGAATTTCAAACTGCA*T*G*T	CATTTTAATGCACCCACTATCTTCAGC	
5	EZH2_2	CTGACTTGTTCACATAACAAACAACTA*T*C*C	AGAACTGTAACCAGTTGCATTTACAAAATC	
6	FLT3_1	GCAGACTGCTGTGAGGGTT*T*T*T	CTCTGGTGTCATTCTTGACAGTGT	
7	FLT3_2_a3	AACTGCCTATTCCTAACTGAC*T*C*A	TTCCAATGGAAAAGAAATGCTGCA	
8	FLT3_3	GAGTGCTCAGTGTCTAATTCCA*C*T*T	ACAGAAAAAGCAGACAGCTCTGAAA	
9	FLT3_4	ACACTGACCCTATACTCTCCTGTA*A*A*A	CACAGAAGGAGTCTGGAATAGAAAGG	
10	FLT3_5_4	TGTTGCTGTCCTTCCACTA*T*A*C	CCATTGGAAAATCTTTAAAATGC	
11	GATA2_1	AGTCTTCGCTTGGGCTT*G*A*T	GGACTCCCTCCCGAGAACTT	
12	IDH1_1	AATGTGTTGAGATGGACGCCT*A*T*T	CTTGTGAGTGGATGGGTAAAACCTAT	
13	IDH2_1_4	CAGAGACAAGAGGATGGCT*A*G*G	GTGGGACCACTATTATCTCTGTCC	
14	JAK2_1	GCAGGTCCATATAAAGGGAC*C*A*A	AGGCATTAGAAAGCCTGTAGTTTTACTTAC	
15	KIT_1	CTAAAATGTGTGATATCCCTAGACAGG*A*T*T	AAATGGTTTTCTTTTCTCCTCCAACCTA	
16	KIT_2	CCTCCTTGTACCTTCCACTC*C*T*T	CTCAGTTCCTGGACAAAAATACCAATCTAT	
17	KRAS_1	AAAGGTGAGTTTGTATTAAAAGGTACT*G*G*T	AAAGAATGGTCCTGCACCAGTAA	
18	KRAS_2	TCCTCATGTACTGGTCCCT*C*A*T	CGTCATCTTTGGAGCAGGAACAAT	
19	NPM1_1_2	TTCTTGGAGTCATATCTTTATCTAG*A*G*T	TCTGCATTATAAAAAGGACAGCC	
20	NRAS_1	ACAACCTAAAACCAACTCTTCCCATA*A*T*T	TGGTGAAACCTGTTTGTTGGACAT	
21	NRAS_2	CACGTTAAGCTTATTGCATAACTGAA*T*G*T	GGTTCTTGCTGGTGTGAAATGAC	
22	PTPN11_1_1	GGTGTTGACTGCGATATTG*A*C*G	CCTGTCCTCCTGCTCAAAAG	
23	PTPN11_2	GCAGCAGACTTTGTGGTCACT*A*A*A	GCCTCCCTTTCCAATGGACTAT	
24	RUNX1_2	CATGGGACTCAGAGTAGAGATA*G*G*T	CGTGGTCCTACGATCAGTCCTA	
25	RUNX1_3	AGTGGGCTCCATCTGGTAC*T*T*A	CCACAATAGGACATCGGCAGAAA	
26	RUNX1_4	CTCAGTGCACAGAAACAAG*C*T*T	CCATCACTGTCTTCACAAACCCA	Reverse primer omitted. Used for antibody capture.
27	RUNX1_5	CGACATGCCGATGCC*G*A*T	CCCATCCTCCTAGGCGGTATC	
28	RUNX1_7	AATTTTGAAATGTGGGTTTG*T*T*G	GTCCTTTGACTGGTGTTTAGGTG	
29	SF3B1_1	GCTATGGTTCATGTTTTGCTTTTACCT*A*A*T	CTTCCATAAAGGCTTTAACACAGAATCAA	
30	SF3B1_2	TGTGTGTGTACCTCTAGTCC*C*A*A	GTGTGCAAAAGCAAGAAGTCCT	
31	SRSF2_2_2	CCTCAGCCCCGTTTA*C*C*T	CTTCGTTCGCTTTCACGAC	
32	TP53_1	GGGTTATAGGGAGGTCAAATAA*G*C*A	GGCCTCTGATTCCTCACTGATTG	
33	TP53_2	TGTGATGAGAGGTGGATGG*G*T*A	CCTCATCTTGGGCCTGTGTTAT	
34	TP53_3	TGCCGTCTTCCAGTTGCTT*T*A*T	CTGCTCACCATCGCTATCTGAG	
35	TP53_4	GGAAAGAGGCAAGGAAAGGTG*A*T*A	GACCTGATTTCCTTACTGCCTCTT	
36	U2AF1_1	GGTGGGTTGGAAGGAGACA*T*T*T	AGTCTTATTAAAGCGTGGATGGCAA	
37	U2AF1_2	AGTCGATCACCTGCCTCAC*T*A*T	GCTCTCATTTTCCCTTACAGAGTCAAC	
38	WT1_1_a2	CCTACCCTAACAAGCTCC*A*G*C	GAACACAGCTGCCAGCAATG	
39	WT1_2	TCCTTCTCTCAACTGAGTCTAAAC*C*T*T	CTCACTGTGCCCACATTGTTAG	
40	WT1_3	GCCTGGAAAAGGAGCTCTT*G*A*A	TCAAGACCTACGTGAATGTTCACATG	
41	chr10_106721610	GCTGACTGCCCTTATTGAG*A*T*G	ACTTTGCCACCTTGATATTATGTTC	
42	chr10_5554293	CCCTAACCATCGTTCCTTC*A*G*G	GGAAACGGGGTGTGCGAA	
43	chr10_77210191	ATGGAGATCAGCTGCTT*G*C*C	TTAACACCGCCTCTCCTGC	
44	chr14_56969005	CAGAGTCCTCTCCAGGGTA*A*G*A	ACCAAATGCAAATACCAGGATGA	
45	chr16_55770629	TCCAGTGCTCCCAGG*C*A*T	GTGGTGAGGAGATCAGGAGGAT	
46	chr16_8569820	ATTTCATGACCACTCTATTTCTT*T*C*T	CATGGACATGGCCTGCAC	
47	chr18_9750662	CGGATTGGCCAGTGCA*T*T*C	TCAGATGAACCAAAGGAAGTATGT	
48	chr6_17076840	TGAACCTAGGAGGCTGA*G*G*T	AGATTCTGGTACATTGTGTCTTTATTCT	
49	chr6_40116264	TGTGTCATGGATCAAGGGT*C*T*T	TCCTTCACCAAATTCTTCCCCG	
50	chr6_62094287	GTTAGCCATTCTCTCTAGT*G*C*C	TCTGCAACTCTACTGATAGTGAT	

Supplementary Table 2: Antibody supplier list and tag sequences

Ab Tag Sequence: /5AzideN/GGCTTGTTGTGATTCGACGATNNNNN[barcode]NNNNNAAGCTTGTTTCTGTGCACTGAG
(N's denote random bases for UMI)

#	Antibody	Supplier	Clone	Catalog No.	Barcode Sequence
1	HLA-DR	BioLegend	L243	307665	CATACAGG
2	CD3	BioLegend	UCHT1	300437	AACGCTTC
3	CD4	BioLegend	OKT4	317403	CGGTTACA
4	CD5	Miltenyi Biotec	UCHT2	130-108-042	CCACTTAG
5	CD7	R&D Systems	848438	MAB7579	GCCAAGTT
6	CD10	Miltenyi Biotec	97C5	130-108-025	TGGCAGAA
7	CD11b	BioLegend	ICRF44	301311	ATGTAGCC
8	CD13	BioLegend	WM15	301723	ACGGAATG
9	CD14	BioLegend	M5E2	301809	TGTGACGT
10	CD15	R&D Systems	ICRF29-2	MAB7368	AACCGAGA
11	CD16	BioLegend	3G8	302049	ACAAGGAC
12	CD19	BioLegend	HIB19	302214	ACTGCCAA
13	CD22	R&D Systems	219934	MAB1968	AAGGTGGT
14	CD30	R&D Systems	81337	MAB229	AGGTCCTA
15	CD33	Miltenyi Biotec	AC104.3E3	130-108-039	GGAACCAT
16	CD34	Miltenyi Biotec	AC136	130-108-040	CAGAGCTA
17	CD38	Miltenyi Biotec	REA671	130-122-288	ACCTCACT
18	CD45	Miltenyi Biotec	5B1	130-108-020	CTAACTCC
19	CD56	Miltenyi Biotec	REA196	130-108-016	CCTTGATC
20	CD64	Miltenyi Biotec	10.1.1	130-108-046	AACAACCG
21	CD71	Miltenyi Biotec	AC102	130-108-043	AGATTCGC
22	CD117	BioLegend	HLDA6	323404	TTCGTTGG
23	CD123	Miltenyi Biotec	AC145	130-108-026	GATGGTCA
24	IgG1 control	BioLegend	MG1-45	401407	AGTCTGTG

Supplementary Table 3: Variants detected in healthy PBMCs

Variant	# Cells Genotyped	Percent of Cells Genotyped	# Cells Mutated (HET or HOM, of Genotyped)	Percent of Cells Mutated (HET or HOM, of Genotyped)	SnpEff Annotation	SnpEff Annotation Impact	SnpEff Protein Change	ClinVar Variation ID	ClinVar Review Status	ClinVar Significance
filter:	**>=10%**	**>=10%**	**>=1%**	**>=1%**						
NRAS:chr1:115256546:T/G	5985	98.5%	88	1.5%	synonymous_variant	LOW	Ile55Ile	-	-	-
SF3B1:chr2:198266943:C/T	5886	96.8%	5886	100.0%	intron_variant	MODIFIER	-	-	-	-
SF3B1:chr2:198267257:G/A	710	11.7%	11	1.5%	intron_variant	MODIFIER	-	-	-	-
IDH1:chr2:209112970:A/G	633	10.4%	9	1.4%	intron_variant	MODIFIER	-	-	-	-
KIT:chr4:55599232:A/C	5984	98.5%	63	1.1%	splice_region_variant&intron_variant	LOW	-	-	-	-
KIT:chr4:55599436:T/C	6031	99.2%	6031	100.0%	intron_variant	MODIFIER	-	-	-	-
NPM1:chr5:170837513:C/T	1128	18.6%	19	1.7%	downstream_gene_variant	MODIFIER	-	-	-	-
LOC101928433-STMND1:chr6:17076799:TA/T	3351	55.1%	2603	77.7%	intergenic_region	MODIFIER	-	-	-	-
LOC101928433-STMND1:chr6:17076799:TAA/T	4138	68.1%	49	1.2%	intergenic_region	MODIFIER	-	-	-	-
LOC101928433-STMND1:chr6:17076799:T/TA	4147	68.2%	464	11.2%	intergenic_region	MODIFIER	-	-	-	-
LOC101928433-STMND1:chr6:17076840:C/A	2734	45.0%	2733	100.0%	intergenic_region	MODIFIER	-	-	-	-
MOCS1-LINC00951:chr6:40116239:A/G	826	13.6%	16	1.9%	intergenic_region	MODIFIER	-	-	-	-
EZH2:chr7:148504716:AG/A	5940	97.7%	4233	71.3%	3_prime_UTR_variant	MODIFIER	-	259400	criteria_provided	Benign
EZH2:chr7:148504854:A/AGACTT	5954	98.0%	5790	97.2%	intron_variant	MODIFIER	-	-	-	-
JAK2:chr9:5073681:CT/C	1676	27.6%	1299	77.5%	splice_region_variant&intron_variant	LOW	-	-	-	-
JAK2:chr9:5073681:C/CT	2336	38.4%	46	2.0%	splice_region_variant&intron_variant	LOW	-	-	-	-
CALML3-AS1:chr10:5554193:A/G	4682	77.0%	47	1.0%	downstream_gene_variant	MODIFIER	-	-	-	-
CALML3-AS1:chr10:5554293:T/C	4280	70.4%	4131	96.5%	downstream_gene_variant	MODIFIER	-	-	-	-
C10orf11:chr10:77210191:C/T	5235	86.1%	5045	96.4%	intron_variant	MODIFIER	-	-	-	-
C10orf11:chr10:77210229:GA/G	3451	56.8%	2626	76.1%	intron_variant	MODIFIER	-	-	-	-
C10orf11:chr10:77210229:G/GA	4345	71.5%	72	1.7%	intron_variant	MODIFIER	-	-	-	-
SORCS3:chr10:106721610:G/A	3005	49.4%	2835	94.3%	intron_variant	MODIFIER	-	-	-	-
WT1:chr11:32417945:T/C	5870	96.6%	5750	98.0%	synonymous_variant	LOW	Arg369Arg	198591	criteria_provided	Benign
PTPN11:chr12:112888251:A/G	1073	17.7%	16	1.5%	synonymous_variant	LOW	Lys89Lys	-	-	-
PTPN11:chr12:112888255:A/G	1074	17.7%	14	1.3%	missense_variant	MODERATE	Lys91Glu	-	-	-
PTPN11:chr12:112927042:T/A	1276	21.0%	383	30.0%	downstream_gene_variant	MODIFIER	-	-	-	-
PTPN11:chr12:112927042:TC/T	1979	32.6%	185	9.3%	downstream_gene_variant	MODIFIER	-	-	-	-
PTPN11:chr12:112927043:C/T	2295	37.8%	2256	98.3%	downstream_gene_variant	MODIFIER	-	-	-	-
FLT3:chr13:28592546:T/C	5777	95.0%	5646	97.7%	intron_variant	MODIFIER	-	-	-	-
FLT3:chr13:28602226:AAGAG/A	4740	78.0%	2784	58.7%	intron_variant	MODIFIER	-	-	-	-
FLT3:chr13:28602226:AAG/A	5287	87.0%	5287	100.0%	intron_variant	MODIFIER	-	-	-	-
FLT3:chr13:28602227:AG/A	4945	81.4%	102	2.1%	intron_variant	MODIFIER	-	-	-	-
FLT3:chr13:28602229:AG/A	5289	87.0%	66	1.2%	intron_variant	MODIFIER	-	-	-	-
PELI2-LOC101927690:chr14:56969005:C/T	985	16.2%	985	100.0%	intergenic_region	MODIFIER	-	-	-	-
IDH2:chr15:90631985:A/G	4864	80.0%	4748	97.6%	splice_region_variant&intron_variant	LOW	-	-	-	-
CES1P2:chr16:55770629:C/T	4651	76.5%	4650	100.0%	intron_variant	MODIFIER	-	-	-	-
TP53:chr17:7578115:T/C	6004	98.8%	6004	100.0%	intron_variant	MODIFIER	-	256603	criteria_provided	Benign
ASXL1:chr20:31022959:T/C	5697	93.7%	5697	100.0%	missense_variant	MODERATE	Leu815Pro	133599	no_assertion_provided	not_provided
U2AF1:chr21:44524505:A/C	4084	67.2%	1869	45.8%	missense_variant	MODERATE	Cys18Gly	-	-	-

Supplementary Table 4: Patient information and treatment histories

Indicates timepoint analyzed by DAb-seq

Gemtuzumab Patient - #1

Patient information

Karyotype:	Normal
WHO 2017 classification:	Acute monoblastic/monocytic leukemia
ELN risk:	Presumed intermediate, FLT3 allelic ratio not available thus not determined
Sequencing at diganosis:	No sequencing performed at diagnosis, NPM1 mutation and FLT3-ITD seen by PCR, CEBPalpha mutation negative by PCR

Treatment history

Months Post-Diagnosis	*Notes*
0	Diagnosis
	Treatment: cytarabine + idarubicin
1	End of induction persistent disease
2	Gilteritinib
3	Persistent disease
4	Treatment: clofarabine, cytarabine
5	Remission
6	HSCT
8	Recurrence
	Treatment: sorafinib + azacitidine x 6
13	Treatment: sorafinib maintenance
51	Relapse
	Treatment: azacitidine + venetoclax
52	End of induction: persistent disease
	Treatment: azacitidine + venetoclax
53	Progressive disease
	Treatment: gemtuzumab + gilteritinib
54	DLI
54	Remission

Key of acronyms

*HSCT= Hematopoetic stem cell transplant
*DLI = donor lymphocye infusion
*FLAG = fludarabine, cytarabine, G-CSF
*ida = idarubicin

Pediatric Patient - #2

Patient information

Karyotype:	47, XY with MLL rearrangement 11q23
WHO 2017 classification:	Not available
ELN risk:	Not available
Sequencing at diganosis:	No sequencing performed at diagnosis, qPCR positive for FLT3 kinase domain mutation at codon 835 or 836 (p.Asp835 or p.Ile836)

Treatment history

Months Post-Diagnosis	*Notes*
0	Diagnosis
	Treatment: per study AAML1031 with bortezomib (cytarabine, daunorubicin, etoposide, bortezomib)
2	Remission
	Treatment: consolidation per AAML1031 (cytarabine, etoposide, mitoxantrone, bortezomib)
10	Relapse

Gilteritinib Patient - #3

Patient information

Karyotype:	46XY
WHO 2017:	AML with mutated NPM1
ELN risk:	Intermediate (NPM1 mutation and FLT3 mutation)
Sequencing at diganosis:	Sequenced with myeloid malignancies panel, mutations in NPM1 (VAF 38.7%), DNMT3A (VAF 41.5%), and IDH2 (VAF 44.9%)

Treatment history

Months Post-Diagnosis	*Notes*
0	Diagnosis
	Treatment: cytarabine/daunorubicin
1	End of induction therapy (persistent NPM1)
2	HDAC + glasdigib x2
3	Persistent NPM1
4	Treatment: azacitidine + venetoclax
5	Recurrence with new FLT3-ITD
6	Treatment: FLAG-Ida
7	Recurrence
	Treatment: gilteritinib
8	Prior to HSCT

Supplementary Table 5: Clinical flow cytometry data

	Timepoint	Blast Fraction	Surface Marker Expression									
			**CD4**	**CD5**	**CD7**	**CD11c**	**CD13**	**CD14**	**CD15**	**CD16**	**CD19**	**CD33**
**Patient 1**	Relapse 2	75.0%	-	-	var		normal	-	-	-	-	+++
	Salvage Therapy	35.0% *	-	-	+ / subs +++		normal	-	-	-	-	+++
	Progression	45.0%	+	n. r.	var +		++	n. r.	n. r.	n. r.	n. r.	+++
	Remission	0.0%	n/a	n/a	n/a		n/a	n/a	n/a	n/a	n/a	n/a
**Patient 2**	Diagnosis	91.0%	++			++	var	-	+		n. r.	++
	Remission	0.0%	n/a			n/a	n/a	n/a	n/a		n/a	n/a
	Relapse	82.0%	++			++	-	-	++		-/+	++
**Patient 3**	Diagnosis	63.0% **	+	n. r.	n. r.		- / +	n. r.	+	n. r.	n. r.	++
	Remission	0.0%	n/a	n/a	n/a		n/a	n/a	n/a	n/a	n/a	n/a
	Relapse	72.0%	+	n. r.	n. r.		++	n. r.	n. r.	n. r.	n. r.	++
	FLT3 Inhibitor	12.6%	-	-	-		+++	-	+	-	-	+++

* second population, ~3.5%: CD123 +++, CD34 -, HLA-DR -
** second population, 1.4%: CD13 ++, CD33 ++, CD34 ++, CD38 ++, CD71 +, CD117 ++, CD123 +, HLA-DR +[illegible]

	Timepoint	Surface Marker Expression (continued)										
		**CD34**	**CD38**	**CD42b**	**CD45**	**CD56**	**CD61**	**CD64**	**CD71**	**CD117**	**CD123**	**HLA-DR**
**Patient 1**	Relapse 2	var	-/+		normal	-		-	normal	var	+++	var
	Salvage Therapy	var / subs +	normal		normal	n. r.		n. r.	normal	var	+++	var / +
	Progression	++ / -	var		n. r.	n. r.		n. r.	+	++	++	++
	Remission	n/a	n/a		n/a	n/a		n/a	n/a	n/a	n/a	n/a
**Patient 2**	Diagnosis	n. r.		-		var	-	++	-	+		++
	Remission	n/a		n/a		n/a	n/a	n/a	n/a	n/a		n/a
	Relapse	-		-		n.r.	-	++	-	++		++
**Patient 3**	Diagnosis	n. r.	n. r.		n. r.	n. r.		++	n. r.	n. r.	+	var
	Remission	n/a	n/a		n/a	n/a		n/a	n/a	n/a	n/a	n/a
	Relapse	n. r.	++		n. r.	n. r.		n. r.	+	++	++	+
	FLT3 Inhibitor	-	+		normal	-		-	normal	normal	+++	+

**Legend**	
+++	*high / bright*
++	*intermediate / expressed*
+	*low / dim*
-	*negative*
var.	*variable*
n. r.	*not reported*
n/a	*not applicable*
subs	*subset with different profile*

Supplementary Table 6: Library preparation primer sequences

Name	Sequence (5' to 3')
Ab P7 Index 1	CAAGCAGAAGACGGCATACGAGATTACTACGCGTGACTGGAGTTCCTTGGCACCCGAGAATTCCAGGCTTGTTGTGATTCG*A*C*G
Ab P7 Index 2	CAAGCAGAAGACGGCATACGAGATAGGCTCCGGTGACTGGAGTTCCTTGGCACCCGAGAATTCCAGGCTTGTTGTGATTCG*A*C*G
Ab P7 Index 3	CAAGCAGAAGACGGCATACGAGATGCAGCGTAGTGACTGGAGTTCCTTGGCACCCGAGAATTCCAGGCTTGTTGTGATTCG*A*C*G
Ab P7 Index 4	CAAGCAGAAGACGGCATACGAGATCTGCGCATGTGACTGGAGTTCCTTGGCACCCGAGAATTCCAGGCTTGTTGTGATTCG*A*C*G
P5 Index 1	AATGATACGGCGACCACCGAGATCTACACTAGATCGCGCCTGTCCGCGGAAGCAGTGGTATCAACGCAG
P5 Index 2	AATGATACGGCGACCACCGAGATCTACACCTCTCTATGCCTGTCCGCGGAAGCAGTGGTATCAACGCAG
P5 Index 3	AATGATACGGCGACCACCGAGATCTACACTATCCTCTGCCTGTCCGCGGAAGCAGTGGTATCAACGCAG
P5 Index 4	AATGATACGGCGACCACCGAGATCTACACAGAGTAGAGCCTGTCCGCGGAAGCAGTGGTATCAACGCAG
P5 Index 5	AATGATACGGCGACCACCGAGATCTACACGTAAGGAGGCCTGTCCGCGGAAGCAGTGGTATCAACGCAG
P5 Index 6	AATGATACGGCGACCACCGAGATCTACACACTGCATAGCCTGTCCGCGGAAGCAGTGGTATCAACGCAG
P5 Index 7	AATGATACGGCGACCACCGAGATCTACACAAGGAGTAGCCTGTCCGCGGAAGCAGTGGTATCAACGCAG
P5 Index 8	AATGATACGGCGACCACCGAGATCTACACCTAAGCCTGCCTGTCCGCGGAAGCAGTGGTATCAACGCAG
P5 Index 9	AATGATACGGCGACCACCGAGATCTACACCGTCTAATGCCTGTCCGCGGAAGCAGTGGTATCAACGCAG
P5 Index 10	AATGATACGGCGACCACCGAGATCTACACTCTCTCCGGCCTGTCCGCGGAAGCAGTGGTATCAACGCAG
P5 Index 11	AATGATACGGCGACCACCGAGATCTACACTCGACTAGGCCTGTCCGCGGAAGCAGTGGTATCAACGCAG
P5 Index 12	AATGATACGGCGACCACCGAGATCTACACTTCTAGCTGCCTGTCCGCGGAAGCAGTGGTATCAACGCAG
P5 Index 13	AATGATACGGCGACCACCGAGATCTACACCCTAGAGTGCCTGTCCGCGGAAGCAGTGGTATCAACGCAG
P5 Index 14	AATGATACGGCGACCACCGAGATCTACACGCGTAAGAGCCTGTCCGCGGAAGCAGTGGTATCAACGCAG
P5 Index 15	AATGATACGGCGACCACCGAGATCTACACCTATTAAGGCCTGTCCGCGGAAGCAGTGGTATCAACGCAG
P5 Index 16	AATGATACGGCGACCACCGAGATCTACACAAGGCTATGCCTGTCCGCGGAAGCAGTGGTATCAACGCAG

Supplementary Table 7: Cost analysis of DAb-seq

Item	Quantity	Unit Cost ($)	Total Purchase Cost ($)	Experiments per Unit	Cost per Experiment ($)
Mission Bio Tapestri cartridge and reagent kit	1	$8,000.00	$8,000.00	8	$1,000.00
Monoclonal antibody (100 µg)	20	$300.00	$6,000.00	50	$120.00
Azide-modified oligonucleotide (100 nmol synthesis)	20	$180.00	$3,600.00	200	$18.00
Sequencing kit (MiSeq v2 300 cycle kit)	1	$800.00	$800.00	1	$800.00
Human TruStain FcX (50 tests)	1	$85.00	$85.00	50	$1.70
Dextran sulfate (10 g)	1	$22.00	$22.00	1000	$0.02
Salmon sperm DNA (5 x 1 mL)	1	$169.00	$169.00	500	$0.34
Ampure XP beads (60 mL)	1	$1,500.00	$1,500.00	50	$30.00
Other labortatory reagents (D-PBS, water, culture media, etc...)	-	$300.00	$300.00	20	$15.00
**TOTAL**			**$20,476.00**		**$1,985.06**

Supplementary Table 8: Genotyping metrics in cell line experiment

Variant (cDNA Coordinates)	Variant (DNA Coordinates)	WT Cell Line	Mutant Cell Line(s)	# of WT Cells Called as WT* [Correct Calls]	# of WT Cells Called as HET or HOM ALT* [Incorrect Calls]	% Incorrect Calls
KIT 2484+78T>C	KIT:chr4:55599436:T/C	Jurkat	Raji, K562	731	148	16.8%
TP53 700T>C	TP53:chr17:7577581:A/G	Raji	Jurkat	776	44	5.4%
TP53 700T>C	TP53:chr17:7577581:A/G	K562	Jurkat	1082	35	3.1%
TP53 586C>T	TP53:chr17:7578263:G/A	K562	Raji	965	50	4.9%
TP53 586C>T	TP53:chr17:7578263:G/A	Jurkat	Raji	814	23	2.7%

*True WT cell population defined by antibody Leiden cluster.
