# Supplementary material for: Joint profiling of DNA and proteins in single cells to dissect genotype-phenotype associations in leukemia
Source: Nat Commun. 2021 Mar 11;12:1583. doi: 10.1038/s41467-021-21810-3 (PMC7952600; doi:10.1038/s41467-021-21810-3)
Supplement: Supplementary file 2 — Reporting Summary [file 41467_2021_21810_MOESM2_ESM.pdf]

nature research

Corresponding author(s): Adam Abate

Last updated by author(s): Feb 2, 2021

Reporting Summary

Nature Research wishes to improve the reproducibility of the work that we publish. This form provides structure for consistency and transparency in reporting. For further information on Nature Research policies, see our Editorial Policies and the Editorial Policy Checklist.

Statistics

For all statistical analyses, confirm that the following items are present in the figure legend, table legend, main text, or Methods section.

n/a	Confirmed	
☐	☒	The exact sample size (*n*) for each experimental group/condition, given as a discrete number and unit of measurement
☐	☒	A statement on whether measurements were taken from distinct samples or whether the same sample was measured repeatedly
☐	☒	The statistical test(s) used AND whether they are one- or two-sided<br>*Only common tests should be described solely by name; describe more complex techniques in the Methods section.*
☒	☐	A description of all covariates tested
☐	☒	A description of any assumptions or corrections, such as tests of normality and adjustment for multiple comparisons
☐	☒	A full description of the statistical parameters including central tendency (e.g. means) or other basic estimates (e.g. regression coefficient) AND variation (e.g. standard deviation) or associated estimates of uncertainty (e.g. confidence intervals)
☐	☒	For null hypothesis testing, the test statistic (e.g. *F*, *t*, *r*) with confidence intervals, effect sizes, degrees of freedom and *P* value noted<br>*Give P values as exact values whenever suitable.*
☒	☐	For Bayesian analysis, information on the choice of priors and Markov chain Monte Carlo settings
☒	☐	For hierarchical and complex designs, identification of the appropriate level for tests and full reporting of outcomes
☒	☐	Estimates of effect sizes (e.g. Cohen's *d*, Pearson's *r*), indicating how they were calculated

Our web collection on statistics for biologists contains articles on many of the points above.

Software and code

Policy information about availability of computer code

Data collection	No software was used for data collection.
Data analysis	All custom code used for analysis is publicly available at www.github.com/AbateLab/DAb-seq. The following dependencies are required (with version numbers in parentheses): GATK (4.1.3.0), bowtie2 (2.3.4.1), ITDseek (1.2), samtools (1.8), bedtools (2.27.1), bcftools (1.9), cutadapt (2.4), BBMap (38.57), snpEff (4.3t). All dependencies are publicly available. The ClinVar database (v.20190805) was used for variant annotation.

For manuscripts utilizing custom algorithms or software that are central to the research but not yet described in published literature, software must be made available to editors and reviewers. We strongly encourage code deposition in a community repository (e.g. GitHub). See the Nature Research guidelines for submitting code & software for further information.

Data

Policy information about availability of data

All manuscripts must include a data availability statement. This statement should provide the following information, where applicable:

- Accession codes, unique identifiers, or web links for publicly available datasets
- A list of figures that have associated raw data
- A description of any restrictions on data availability

All sequencing data generated in this study is available on the Sequence Read Archive under the BioProject with accession number PRJNA602320.

Field-specific reporting

Please select the one below that is the best fit for your research. If you are not sure, read the appropriate sections before making your selection.

☒ Life sciences ☐ Behavioural & social sciences ☐ Ecological, evolutionary & environmental sciences

For a reference copy of the document with all sections, see nature.com/documents/nr-reporting-summary-flat.pdf

Life sciences study design

All studies must disclose on these points even when the disclosure is negative.

Sample size	No explicit calculations were performed to determine sample size. Rather, we aimed to capture multiple treatment timepoints from individual patients with different disease histories. Therefore, we investigated 3 patients at 11 total timepoints. We observed a total of 54,717 cells after filtering (see Data Exclusions). The total number of cells analyzed is consistent with typical throughput of the Tapestri instrument and was not pre-determined.
Data exclusions	Cell barcodes likely representing background noise rather than true cells were excluded as described in the online methods. To be considered valid, barcodes were required to have a minimum of 100 antibody UMIs by the adjacency counting method and a maximum IgG1 count no greater than five times the median IgG1 count of the associated DAb-seq experiment. The exclusion criteria were pre-established and are consistent with current practices in single-cell sequencing. These criteria were applied to all experiments.
Replication	Due to limited availability of patient tissue samples, each timepoint was analyzed in a single DAb-seq experiment. However, the longitudinal nature of our study (featuring 3 or more timepoints per patient) allowed us to confirm that healthy and diseased blood cell populations present in multiple collections had consistent mutational and immunophenotypic signatures. The reproducibility of protein and DNA measurements is shown in the article figures and Supplementary Figure 13, where persistent cell populations co-localize in the same proteogenomic clusters.
Randomization	Randomization was not relevant for this study. All samples were processed and analyzed identically, regardless of patient clinical history or other external criteria.
Blinding	Blinding was not relevant for this study. Sample labels were required to associate DAb-seq data with corresponding flow cytometry and bulk genotyping data, and assess the extent of agreement between the measurements.

Reporting for specific materials, systems and methods

We require information from authors about some types of materials, experimental systems and methods used in many studies. Here, indicate whether each material, system or method listed is relevant to your study. If you are not sure if a list item applies to your research, read the appropriate section before selecting a response.

Materials & experimental systems

n/a	Involved in the study
☐	☒ Antibodies
☐	☒ Eukaryotic cell lines
☒	☐ Palaeontology and archaeology
☒	☐ Animals and other organisms
☐	☒ Human research participants
☒	☐ Clinical data
☒	☐ Dual use research of concern

Methods

n/a	Involved in the study
☒	☐ ChIP-seq
☐	☒ Flow cytometry
☒	☐ MRI-based neuroimaging

Antibodies

Antibodies used	A full list of antibodies, clone, supplier and catalog number is provided in Supplementary Table 2. For all experiments, 0.5 micrograms of each individual antibody-oligonucleotide conjugate was used.
Validation	For all antibodies, experimental validation data and quality certificates are provided on the commercial supplier's website (see Supplementary Table 2 for manufacturer information and catalog number). All antibodies were validated for use in flow cytometry applications using cells known to express the relevant surface markers.

Eukaryotic cell lines

Policy information about cell lines

Cell line source(s)	The following cell lines were used in the study: Raji (ATCC, CCL-86), Jurkat (ATCC, TIB-152), K562 (ATCC, CCL-243). All cell lines were acquired directly from the American Type Culture Collection (ATCC).
Authentication	Cell lines were ordered directly from and authenticated by the ATCC. ATCC performs morphology, karyotyping, and PCR-based approaches for cell line authentication. This includes STR profiling and COI analysis. Furthermore, our own DNA genotyping confirmed the presence of cell type-specific DNA polymorphisms.
Mycoplasma contamination	Cell lines tested as negative for mycoplasma contamination.
Commonly misidentified lines (See ICLAC register)	No commonly misidentified cell lines were used.

Human research participants

Policy information about studies involving human research participants

Population characteristics	The study included cryopreserved bone marrow or peripheral blood samples from patients with documented acute myeloid leukemia (n = 3). Patients underwent therapy as indicated at the discretion of the treating physician. Written informed consent was obtained from all patients. Supplementary Table 4 provides a summary of clinical histories for each patient. Age and gender were not treated as covariates in this study and are not reported.
Recruitment	Samples analyzed in this study were from patients being treated at University of California, San Francisco (UCSF). Patient samples were selected for analysis based on availability of banked tissue across multiple treatment timepoints. Therefore, patients for whom samples were not available at multiple timepoints were not considered for inclusion in this study. These selection criteria are not expected to bias the outcome or impact the claims made in the study.
Ethics oversight	Sample collection was in accordance with the Declaration of Helsinki under tissue banking protocols approved by the UCSF Committee on Human Research, which is UCSF's Institutional Review Board. Written informed consent was obtained from all patients.

Note that full information on the approval of the study protocol must also be provided in the manuscript.

Flow Cytometry

Plots

Confirm that:

- [x] The axis labels state the marker and fluorochrome used (e.g. CD4-FITC).
- [x] The axis scales are clearly visible. Include numbers along axes only for bottom left plot of group (a 'group' is an analysis of identical markers).
- [x] All plots are contour plots with outliers or pseudocolor plots.
- [x] A numerical value for number of cells or percentage (with statistics) is provided.

Methodology

Sample preparation	Samples for flow cytometry were processed as described in the Methods section of the manuscript.
Instrument	BD FACSAria II
Software	FlowJo v10 was used to analyze the flow cytometry data.
Cell population abundance	No cell sorting was performed in this study.
Gating strategy	The FSC/SSC scatter was gated on singlets. Remaining gates were drawn between the negative and positive populations for each marker to serve as a basis for comparison with the DAb-seq data.

- [x] Tick this box to confirm that a figure exemplifying the gating strategy is provided in the Supplementary Information.
